# Hypertension and incident cardiovascular events after next-generation BTKi therapy initiation

**DOI:** 10.1186/s13045-022-01302-7

**Published:** 2022-07-14

**Authors:** Sunnia T. Chen, Leylah Azali, Lindsay Rosen, Qiuhong Zhao, Tracy Wiczer, Marilly Palettas, John Gambril, Onaopepo Kola-Kehinde, Patrick Ruz, Sujay Kalathoor, Kerry Rogers, Adam Kittai, Michael Grever, Farrukh Awan, John C. Byrd, Jennifer Woyach, Seema A. Bhat, Daniel Addison

**Affiliations:** 1 Cardio-Oncology Program, Division of Cardiology, The Ohio State University Medical Center, Columbus, OH USA; 2 Department of Pharmacy, James Cancer Hospital and Solove Research Institute at The Ohio State University, Columbus, OH USA; 3 Center for Biostatistics, Department of Biomedical Informatics, The Ohio State University, Columbus, OH USA; 4 Division of Hematology, The Ohio State University, Columbus, OH USA; 5 Division of Hematology, UT-Southwestern, Dallas, TX USA; 6 Department of Medicine, University of Cincinnati, Cincinnati, OH USA; 7 Division of Cancer Prevention and Control, Department of Internal Medicine, College of Medicine, The Ohio State University, Columbus, OH USA; 8 Division of Cardiovascular Medicine, Davis Heart & Lung Research Institute, 473 West 12th Avenue, Suite 200, Columbus, OH 43210 USA

**Keywords:** Hypertension, Cardiovascular events, Acalabrutinib, Cancer-targeted therapy, Cardio-oncology

## Abstract

**Background:**

Post-market analyses revealed unanticipated links between first-generation Bruton’s tyrosine kinase inhibitor (BTKi) therapy, ibrutinib, and profound early hypertension. Yet, whether this is seen with novel selective second (next)-generation BTKi therapy, acalabrutinib, is unknown.

**Methods:**

Leveraging a large cohort of consecutive B cell cancer patients treated with acalabrutinib from 2014 to 2020, we assessed the incidence and ramifications of new or worsened hypertension [systolic blood pressure (SBP) ≥ 130 mmHg] after acalabrutinib initiation. Secondary endpoints were major cardiovascular events (MACE: arrhythmias, myocardial infarction, stroke, heart failure, cardiac death) and disease progression. Observed incident hypertension rates were compared to Framingham heart-predicted and ibrutinib-related rates. Multivariable regression and survival analysis were used to define factors associated with new/worsened hypertension and MACE, and the relationship between early SBP increase and MACE risk. Further, the effect of standard antihypertensive classes on the prevention of acalabrutinib-related hypertension was assessed.

**Results:**

Overall, from 280 acalabrutinib-treated patients, 48.9% developed new/worsened hypertension over a median of 41 months. The cumulative incidence of new hypertension by 1 year was 53.9%, including 1.7% with high-grade (≥ 3) hypertension. Applying the JNC 8 cutoff BP of ≥ 140/90 mmHg, the observed new hypertension rate was 20.5% at 1 year, > eightfold higher than the Framingham-predicted rate of 2.4% (RR 8.5, *P* < 0.001), yet 34.1% lower than ibrutinib (12.9 observed-to-expected ratio, *P* < 0.001). In multivariable regression, prior arrhythmias and Black ancestry were associated with new hypertension (HR 1.63, HR 4.35, *P* < 0.05). The degree of SBP rise within 1 year of treatment initiation predicted MACE risk (42% HR increase for each + 5 mmHg SBP rise, *P* < 0.001). No single antihypertensive class prevented worsened acalabrutinib-related hypertension.

**Conclusions:**

Collectively, these data suggest that hypertension may be a class effect of BTKi therapies and precedes major cardiotoxic events.

**Supplementary Information:**

The online version contains supplementary material available at 10.1186/s13045-022-01302-7.

## Introduction

Acalabrutinib is a novel selective, second-generation Bruton’s tyrosine kinase (BTK) inhibitor with dramatic efficacy against B cell malignancies [1–5]. Compared to ibrutinib, a first in-class BTK inhibitor, acalabrutinib has lower alternative kinase inhibition and off-target activity [6–8]. Due to a high burden of cardiotoxicity, namely the development of new or worsened hypertension or arrhythmias in follow-up, indefinite ibrutinib use has been limited to those without intolerable effects or disease progression [9–16]. In available initial clinical trials, acalabrutinib associates with similar efficacy, but lower rates of adverse events [3, 17, 18].

However, with ibrutinib, post-trial clinical and disproportionality analyses revealed signals of higher cardiovascular event risks, often preceded by incident hypertension, not observed in initial clinical trials [11–16]. With acalabrutinib, available secondary analyses have suggested lower arrhythmia risks, but conflicting rates for hypertension and other cardiovascular events [1, 3–5, 17]. In ACE-CL-001, no cases of high-grade hypertension were reported^1^, while in ELEVATE-RR, 9% developed hypertension^18^; and in a recent Phase 1/2a trial, up to 40% of patients treated with acalabrutinib developed incident hypertension (any-grade) [4]. However, to date, there are no data on the relationship of acalabrutinib to hypertension or other cardiovascular risks in routine clinical practice. Given the insidious nature of blood pressure elevation, and its potential relationship to longer-term cardiotoxicity risk [11], understanding these effects may prove pivotal. Yet, whether early hypertension is also seen with acalabrutinib or holds consequences for the risk of subsequent major cardiovascular events in during treatment is unknown.

## Methods

### Study population

From a large US-based Comprehensive Cancer Center cohort of consecutive patients initiated on acalabrutinib from 2014 to 2020, we assessed the incidence of new or worsened hypertension, after Institutional Review Board approval. Study patients included adults ≥ 18 years of age treated with acalabrutinib for any lymphoid malignancy. Blood pressure and other traditional cardiovascular variables were collected across time. Patients with incomplete medical records for the cancer and cardiovascular variables of interest were excluded. Incident (new) hypertension was defined as systolic blood pressure (SBP) ≥ 130 mmHg on two separate visits within 3 months, accounting for contemporary hypertension definitions after publication of the Systolic Blood Pressure Interventional Trial (SPRINT) and the stronger correlation between change in SBP (compared with diastolic blood pressure) and major cardiovascular events after 50 years of age [19, 20]. Worsened hypertension was defined as an increase in hypertension grade by Common Terminology Criteria for Adverse Events (CTCAE) v5.0 or an increase in antihypertensive therapy [21]. The presence of baseline hypertension was considered to be a documented SBP ≥ 130 mmHg on two visits within 3 months before acalabrutinib initiation or a reported history of hypertension with the current use of at least one blood pressure lowering medication [20, 22]. Baseline antihypertensive therapy use by medication class was also recorded. Furthermore, we also manually identified all major cardiovascular events (MACE) after acalabrutinib initiation, inclusive of incident or recurrent atrial fibrillation (AF), ventricular arrhythmias, heart failure, myocardial infarction, stroke, and sudden cardiac death.

### Outcomes

Our primary outcome was the incidence of new or worsened hypertension after acalabrutinib initiation. The secondary outcome was the occurrence of MACE during acalabrutinib use. Follow-up began from time of acalabrutinib initiation. Hypertension severity and MACE were graded using CTCAE v5.0 and then adjudicated by two independent cardiologists. A Naranjo Probability Score was also calculated for new or worsened hypertension, as well as MACE, to determine the likelihood of acalabrutinib association, with a score of ≥ 5 suggestive of at least probable association [23]. Furthermore, we explored the effects of baseline antihypertensive use on the avoidance and control of new or worsened hypertension development across time.

### Statistical analysis

Descriptive statistics were used to summarize patient characteristics, using mean ± standard deviation (SD) or median (interquartile range, IQR) for continuous variables, and frequency counts with percentages for categorical variables. Univariate and multivariable modeling was used to determine the association between baseline covariates and new or worsened hypertension development. Time-to-event analysis methods were used to assess the association of patient specific factors with hypertension, as well as subsequent cardiac events. The relationship between hypertension development and cardiac events was also assessed using estimated hazard ratios (HR). For each analysis, acalabrutinib discontinuation or death without the outcome of interest was considered as competing risks. Patients without competing events or the outcome of interest were considered censored at the last follow-up date. Cumulative incidence estimates for the primary outcomes were estimated, and cumulative incidence curves were subsequently generated. Events per person-years of follow-up were also assessed, by new or worsened hypertension status.

Traditional risk factors identified through univariable modeling were chosen for multivariable modeling. Fine and gray proportional sub-distribution hazard regression was used, accounting for competing risk of acalabrutinib discontinuation or death. In addition to factors significant on univariate modeling, traditional risk factors including age, smoking status, diabetes, chronic kidney disease (CKD), sex, Black race, and body mass index (BMI) were included in the multivariate model as control variables regardless of the univariate P value observed. All variables with a *P* < 0.10 in univariate modeling were initially included in the multivariable models, and backward selection was used to sequentially (stepwise) remove variables with *P* > 0.05 from the final model. A similar modeling approach was applied to MACE, with the primary comparison of patients who did not have an increase in CTCAE hypertension grade and did not require additional antihypertensive therapy during follow-up. Covariates in the new or worsened hypertension vs. no or stable hypertension models for the occurrence of post-acalabrutinib MACE included age, sex, diabetes, CKD, BMI, coronary artery disease, prior heart failure, AF, and stroke.

Further, we assessed the relationship of peak SBP increase within 1 year of acalabrutinib initiation to risk of developing MACE, inclusive of arrhythmias, using a log-rank test for trend. Hazard ratios were stratified by peak SBP increase. We also assessed the risk of MACE and AF, respectively, in relation to overt new or worsened hypertension after acalabrutinib initiation. In sensitivity analyses, new or worsened hypertension status was considered to be a time-varying covariate: Patients were classified as no or stable hypertension up to the time point of new or worsened hypertension development and remained in the new or worsened hypertension category from that point forward. Additionally, we assessed the effects of antihypertensive therapy initiation on the prevention of subsequent MACE, inclusive of monotherapy or the need for combinational treatment. We did not consider patients initiated on antihypertensive therapies (ex. beta-blockers) for the management of MACE after acalabrutinib initiation, to minimize confounding reasons for therapy initiation. In addition, to further assess the effects of hypertension on disease outcomes, we performed a landmark analysis for differences in cancer disease progression or death, inclusive of only patients surviving 90 days without disease progression or acalabrutinib discontinuation.

To better understand acalabrutinib’s effects on blood pressure elevation, observed rates of incident hypertension (at ≥ 140/90 mmHg) were compared to Framingham-predicted (expected) rates at 1 year post-therapy initiation [24, 25]. Within this, only those with patients aged 20 to 69 years without a diagnosis of diabetes were included, akin to the originally validated prediction model.^24^ The observed rates of hypertension were calculated using the cumulative incidence of blood pressure ≥ 140/90 mmHg at 3-month intervals across the initial year of treatment. The Framingham-predicted rate of new hypertension within 1 year was estimated by averaging the individual patient probabilities obtained after applying individual patient-level risk factors. All analyses were performed with R version 3.6.2 and SAS Software version 9.4, and the statistical tests were two-sided with statistical significance evaluated at the *α* = 0.05 significance level.

## Results

Overall, 280 patients treated with acalabrutinib were identified (Additional file 1: Figure S1). The mean age was 63.7 ± 10.1 years (range 20–89 years), and 28.9% of patients were female. Most (89.0%) had chronic lymphocytic leukemia (CLL), and 279 had an Eastern Cooperative Oncology Group (ECOG) performance status of 0–2. Seventy-two patients were previously treated with ibrutinib; 165 (58.9%) had baseline hypertension at the time of acalabrutinib initiation, of which 57% were on at least 1 antihypertensive medication. Additional baseline characteristics are described in Table 1.

### Incident hypertension

**Fig. 1:**
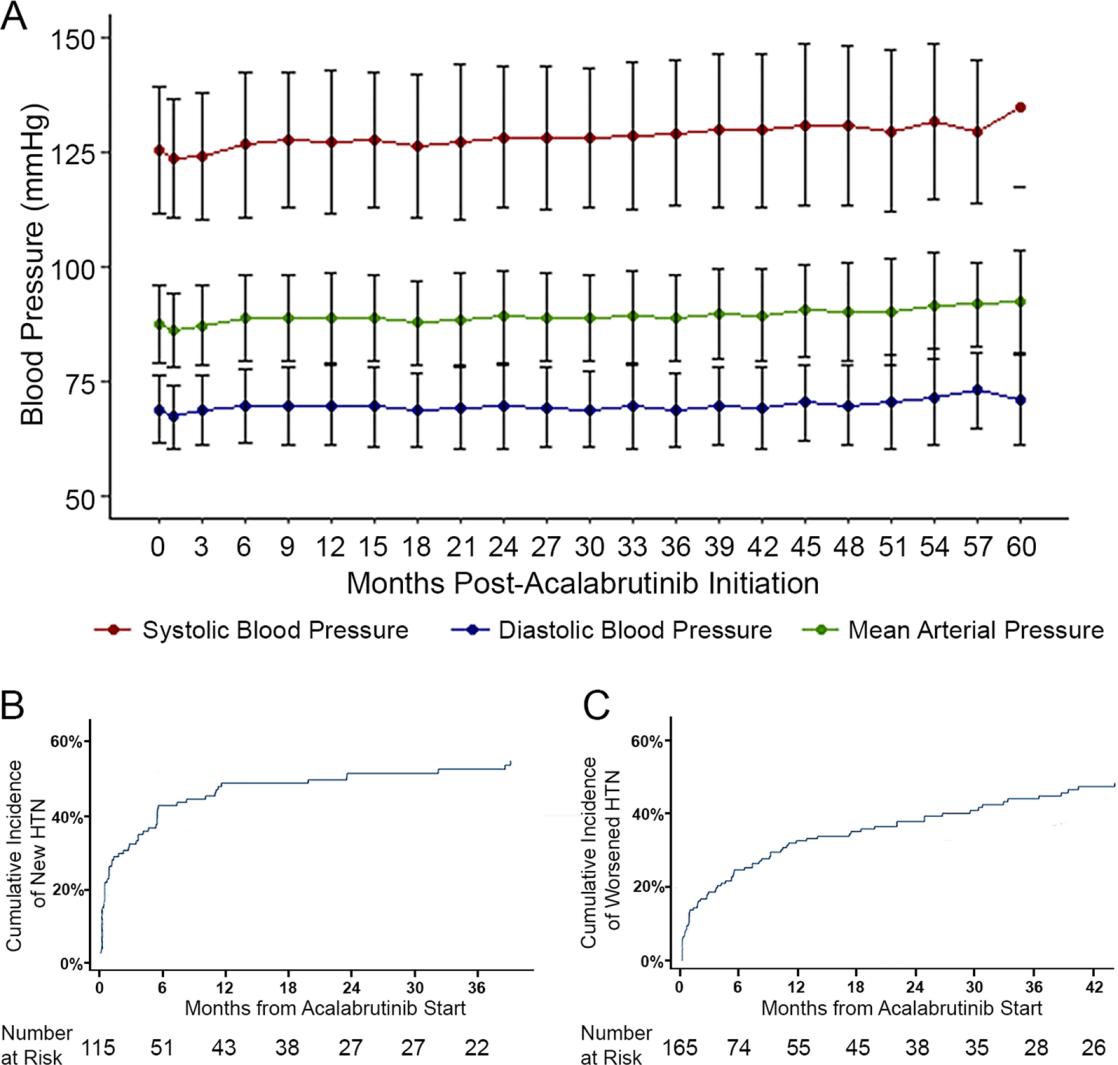
**A**. Change in mean blood pressure during acalabrutinib treatment over the 60-month study period; standard deviation represented by error bars. **B**. Cumulative incidence of new hypertension (HTN) across time following acalabrutinib initiation. **C**. Cumulative incidence of worsened HTN across time following acalabrutinib initiation

Over a median follow-up of 41 months (IQR 20–62 months; range 0–76 months), 59.2% developed new or worsened hypertension, utilizing a SBP cutoff of 130 mmHg (Fig. 1; Additional file 1: Table S1), of which 84.3% had at least probable association with acalabrutinib [23]. The mean increase in SBP was + 7.2 (19.7) mmHg, with a median time to maximum SBP increase of 6 months. A ≥ 10 mmHg increase in SBP from baseline was observed in 35% of patients, and a ≥ 20 mmHg increase was observed in 13.5% of patients (Additional file 1: Table S2). Among those without baseline hypertension, 62 patients (53.9%) developed new hypertension after acalabrutinib initiation, wherein the mean increase in SBP (SD) was 16.7 mmHg (24.2), with a median time to maximum SBP increase of 15 months. Within 1 year of follow-up, the mean maximum increase in SBP was 14.4 mmHg, with a median time to maximum increase in SBP of 6 months, in those with new hypertension. This included 82.3% (*n* = 51) who reached hypertension thresholds within 6 months of acalabrutinib initiation. In those with baseline hypertension, worsened hypertension was noted among 45.5% (*n* = 75), including 50.7% (*n* = 38) with an increase in CTCAE hypertension grade. Moreover, among those treated without intervening monoclonal antibody therapy (obinutuzumab, a known blood pressure depressant), the mean increase in SBP (SD) was 6.45 mmHg (20.0) and the mean peak increase in SBP was 10.1 mmHg, with a median time to maximum SBP increase of 6 months (Additional file 1: Table S3). In total, 9 (3.5%, excluding 20 with baseline high-grade hypertension) developed new high-grade (≥ 3) hypertension while on acalabrutinib, including 1.7% of those without baseline hypertension. No patients required dose reduction due to hypertension. One patient required hospitalization for worsened hypertension (and diastolic heart failure).

In univariate analysis, BMI > 25, prior arrhythmia, and history of heart failure were associated with new or worsened hypertension (Additional file 1: Table S4). There was no relationship between acalabrutinib dose and new or worsened hypertension development. However, in multivariable modeling, Black ancestry [hazard ratio (HR) 4.35, 95% confidence interval (CI) 1.21–15.63; *P* = 0.024], prior AF (HR 1.63, 95% CI 1.06–2.49; *P* = 0.025), and BMI (HR 1.05, 95% CI 1.02–1.09; *P* = 0.005) remained associated with new or worsened hypertension (Table 2). These effects were consistent even after removing patients previously on ibrutinib to account for possible confounding of prior AF (Additional file 1: Table S5A). Among patients without baseline hypertension, Black ancestry and prior arrhythmias were significantly associated with development of new hypertension (Additional file 1: Table S5B); additionally, there was a trend of association between baseline SBP and new hypertension development (HR 1.75 across baseline SBP strata vs SBP ≥ 120 mmHg; *P* = 0.126). Among those with baseline hypertension, only age and BMI were associated with worsened hypertension development (Additional file 1: Table S5C).

**Table 1:** Baseline characteristics. The median duration of acalabrutinib use was 41.2 months

Variable	Total (*n* = 280)	No baseline HTN (*n* = 115)	Baseline HTN (*n* = 165)
Age at acalabrutinib initiation, mean (SD)	63.7 (10.1)	61.5 (10.9)	65.2 (9.3)
Sex, *n* (%)			
Male	199 (71.1)	79 (68.7)	120 (72.7)
Female	81 (28.9)	36 (31.3)	45 (27.3)
Race, *n* (%)			
White	270 (96.8)	111 (96.5)	159 (97.0)
Black	5 (1.8)	3 (2.6)	2 (1.2)
Other*	5 (1.8)	1 (0.9)	4 (2.4)
BMI, mean (SD)	28.1 (5.5)	26.2 (4.5)	29.5 (5.8)
BMI, *n* (%)			
< 25	78 (27.9)	43 (37.4)	35 (21.2)
25–29.9	116 (41.4)	53 (46.1)	63 (38.2)
≥ 30	86 (30.7)	19 (16.5)	67 (40.6)
Other baseline traditional HTN risk factors			
DM	24 (8.6)	9 (7.8)	15 (9.1)
MI	12 (4.3)	4 (3.5)	8 (4.8)
CKD	7 (2.5)	3 (2.6)	4 (2.4)
CHF	12 (4.3)	4 (3.5)	8 (4.8)
AF/Aflutter	40 (14.3)	16 (13.9)	24 (14.5)
CVA/TIA	7 (2.5)	3 (2.6)	4 (2.4)
Smoking status			
Never	166 (59.3)	79 (68.7)	87 (52.7)
Previous	93 (33.2)	27 (23.5)	66 (40.0)
Current	21 (7.5)	9 (7.8)	12 (7.3)
Primary malignancy, *n* (%)			
CLL	249 (88.9)	104 (90.4)	145 (87.9)
MCL	6 (2.1)	2 (1.7)	4 (2.4)
Other†	25 (8.9)	9 (7.8)	16 (9.7)
RAI stage, *n* (%)**			
0	0 (0.0)	0 (0.0)	0 (0.0)
1	43 (15.4)	20 (17.4)	23 (13.9)
2	46 (16.4)	18 (15.7)	28 (17.0)
3	27 (9.6)	15 (13.0)	12 (7.3)
4	90 (32.1)	38 (33.0)	52 (31.5)
Unknown	74 (26.4)	24 (20.9)	50 (30.3)
Baseline ECOG performance status, *n* (%)			
0	107 (38.2)	43 (37.4)	64 (38.8)
1	164 (58.6)	68 (59.1)	96 (58.2)
2	8 (2.9)	3 (2.6)	5 (3.0)
3	1 (0.4)	1 (0.9)	0 (0.0)
4	0 (0.0)	0 (0.0)	0 (0.0)
Unknown	0 (0.0)	0 (0.0)	0 (0.0)
Treatment history, *n* (%)			
Number of prior anticancer therapies, median (IQR)	2 (2)	2 (3)	2 (2)
Concomitant chemotherapy	99 (35.4)	42 (36.5)	57 (34.5)
Prior chemotherapy	137 (48.9)	53 (46.1)	84 (50.9)
Prior monoclonal antibody	171 (61.1)	72 (62.6)	99 (60.0)
Prior ibrutinib therapy	72 (25.7)	26 (22.6)	46 (27.9)
Prior targeted therapy (not Ibrutinib)	26 (9.3)	12 (10.4)	14 (8.5)
Prior immunomodulatory	31 (11.1)	16 (13.9)	15 (9.1)
CY3PA4 inhibitor	27 (9.6)	8 (7.0)	19 (11.5)
Cyclosporine during ibrutinib use	4 (1.4)	1 (0.9)	3 (1.8)
No prior anticancer therapies, *n* (%)	77 (27.5)	33 (28.7)	44 (26.7)
Baseline SBP, mmHg			
< 100	6 (2.1)	6 (5.2)	0 (0.0)
100–119	75 (26.8)	73 (63.5)	2 (1.2)
120–129	68 (24.3)	36 (31.3)	32 (19.4)
130–139	50 (17.9)	0 (0.0)	50 (30.3)
140–179	78 (27.9)	0 (0.0)	78 (47.3)
180 +	3 (1.1)	0 (0.0)	3 (1.8)
Baseline DBP, mmHg			
< 70	124 (44.3)	78 (67.8)	46 (27.9)
70–79	101 (36.1)	33 (28.7)	68 (41.2)
80–89	44 (15.7)	4 (3.5)	40 (24.2)
90–119	11 (3.9)	0 (0.0)	11 (6.7)
Baseline anti-HTN medications			
Beta-blocker	67 (23.9)	25 (21.7)	42 (25.5)
ACE inhibitor/ARB	80 (28.6)	21 (18.3)	59 (35.8)
Calcium channel blocker	29 (10.4)	5 (4.3)	24 (14.5)
Diuretic^‡^	46 (16.4)	11 (9.6)	35 (21.2)
Other^§^	10 (3.5)	2 (1.7)	8 (4.8)

*ACE* angiotensin-converting enzyme inhibitor, *AF* atrial fibrillation, *Aflutter* atrial flutter, *ARB* angiotensin receptor blocker, *BMI* body mass index, *BP* blood pressure, *CKD* chronic kidney disease, *CLL* chronic lymphocytic lymphoma, *CVA* cerebrovascular accident, *CY3PA4* cytochrome P450, family 3, subfamily A, *DBP* diastolic blood pressure, *DM* diabetes mellitus, *ECOG* Eastern Cooperative Oncology Group, *HTN* hypertension, *MCL* mantle cell lymphoma, *MI* myocardial infarction, *TIA* transient ischemic attack, *WM* Waldenström’s macroglobulinemia

^*^Hispanic, Asian, multiracial, and unknown race. **CLL alone. †Diffuse large B cell lymphoma, follicular lymphoma, hairy cell leukemia, graft-versus-host disease, and marginal zone lymphoma. ^‡^Includes loop, thiazide, and potassium-sparing diuretics. §Clonidine, hydralazine, nitrates, and alpha-1 antagonists

**Table 2:** Multivariable predictors for the development of new or worsened hypertension (HTN) (*n* = 280)

Variable	Hazard ratio	95% Confidence interval	*p* value
Age*	1.02	(1.00 – 1.04)	0.078
Sex: Female vs. Male	0.59	(0.37 – 0.92)	**0.021**
Black/African-American	4.35	(1.21 – 15.63)	**0.024**
BMI*	1.05	(1.01 – 1.08)	**0.005**
Smoking status: Current/Previous vs. Never	0.71	(0.50 – 1.01)	0.057
Prior DM	1.38	(0.71 – 2.69)	0.339
Prior CKD	0.58	(0.12 – 2.77)	0.496
Prior AF/AFlutter	1.63	(1.06 – 2.49)	**0.025**
Hematologic diagnosis			
CLL	Reference	Reference	
MCL	1.15	(0.34 – 3.96)	0.82
Other^‡^	0.22	(0.07 – 0.65)	**0.006**
Number of prior anticancer therapies	0.87	(0.78 – 0.97)	**0.015**
Baseline SBP by baseline HTN status interaction	0.99	(0.98 – 0.99)	**0.001**†

Bold indicates statistical significance, using the significance level α = 0.05

*AF* atrial fibrillation, *Aflutter* atrial flutter, *BMI* body mass index, *CKD* chronic kidney disease, *DM* diabetes mellitus, *SBP* systolic blood pressure. *Considered a continuous variable. †Omnibus *p* value (reflects overall variable effect). ‡Diffuse large B cell lymphoma, follicular lymphoma, hairy cell leukemia, graft-versus-host disease, marginal zone lymphoma, and Waldenström’s macroglobulinemia.

#### Effect of standard antihypertensive therapies

The initiation of antihypertensive therapy was required for 43 patients, including 10 without baseline hypertension. Over time, 17 required combination therapy for the management of hypertension after acalabrutinib initiation. The most common treatment was diuretics, followed by calcium channel blockers and beta-blockers. There was no difference in effect of any specific class on blood pressure control (Additional file 1: Table S6A-C). Yet, the initiation of combination therapy was associated with a –3.32 mmHg reduction in SBP over time (Additional file 1: Table S6D).

#### Relationship of new or worsened hypertension to major cardiovascular events

**Fig. 2:**
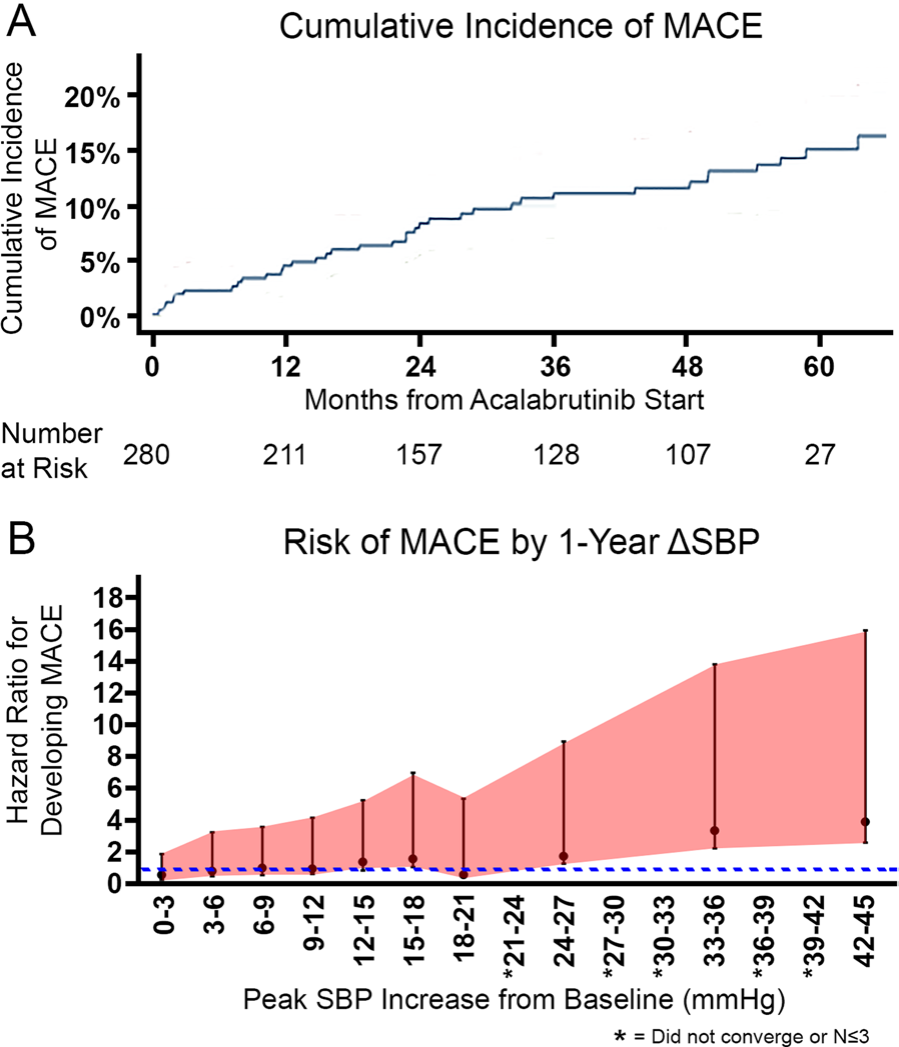
**A** Cumulative incidence of major cardiovascular events (MACE) across time following acalabrutinib initiation. **B**. Risk of developing MACE defined by hazard ratio (HR) stratified by peak systolic blood pressure (SBP) increase within 1 year of acalabrutinib initiation. Dotted line represents hazard ratio = 1. *Asterisks denote statistical tests that did not converge or strata with ≤ 3 patients

MACE were observed among 41 patients (14.6%), including 18.2% with new or worsened hypertension. This was compared to 11.2% with MACE among those without new or worsened hypertension after acalabrutinib initiation (Additional file 1: Table S7). Most events, including 62.1% of arrhythmias, were of probable or definite association with acalabrutinib [23]. AF was the most common cardiovascular complication during acalabrutinib use (8.2%), followed by ventricular arrhythmia (2.9%), SVT (2.5%), and heart failure (2.1%); 1 patient had sudden death. In those with MACE, 66.7% had at least two cardiac risk factors. There was no clear difference in MACE by new or worsened hypertension status in a multivariable model containing known predictors of MACE (HR 1.12, *P* = 0.751; Table 3; Additional file 1: Table S8A-B). However, the magnitude of early SBP increases within 1 year of acalabrutinib initiation related to the risk of subsequent AF (*P* < 0.001; Additional file 1: Figure S2), as well as MACE (*P* < 0.001; Fig. 2). For every 5 mmHg SBP increase, there was a 27% (0.27) increase in MACE risk (*P* < 0.001), and a 42% (0.42) increase in the risk for AF development (*P* < 0.001). Among those with new or worsened hypertension, there was no difference in MACE risk by antihypertensive initiation status. Further, in landmark analysis restricted to those free of disease progression with continued acalabrutinib use beyond 3 months, there was no difference in progression-free survival or mortality by new or worsened hypertension status (Additional file 1: Figure S3).

**Table 3:** Multivariable predictors of major adverse cardiovascular events (MACE) during acalabrutinib use.*

Variable	Hazard ratio	95% CI	*p* value
New/Worsened HTN versus No/Stable HTN**	1.13	(0.52 – 2.46)	0.759
Age ≥ 65	2.07	(1.02 – 4.18)	**0.043**
Sex: Female vs. Male	0.51	(0.20 – 1.26)	0.143
Number of prior anticancer therapies	1.05	(0.91 – 1.21)	0.521
Prior DM	0.94	(0.32 – 2.76)	0.905
Prior CKD	1.11	(0.22 – 5.63)	0.903
Prior AF/Aflutter	3.66	(1.68 – 7.94)	**0.001**
Prior CVA/TIA	2.60	(0.58 – 11.63)	0.212
Prior targeted agent therapies (not ibrutinib)	1.45	(0.54 – 3.88)	0.452

Bold indicates statistical significance, using the significance level α = 0.05

*AF* atrial fibrillation, *Aflutter* atrial flutter, *BMI* body mass index, *CI* confidence interval, *CKD* chronic kidney disease, *CVA* cerebrovascular accident, *DM* diabetes mellitus, *HTN* hypertension, *TIA* transient ischemic attack. *MACE includes the combined outcome of AF, CHF, CVA, MI (myocardial infarction), VF/VT (ventricular fibrillation/ventricular tachycardia), and cardiovascular death during acalabrutinib use. **HTN as non-time-varying

### Comparative incidence of hypertension with acalabrutinib

**Fig. 3:**
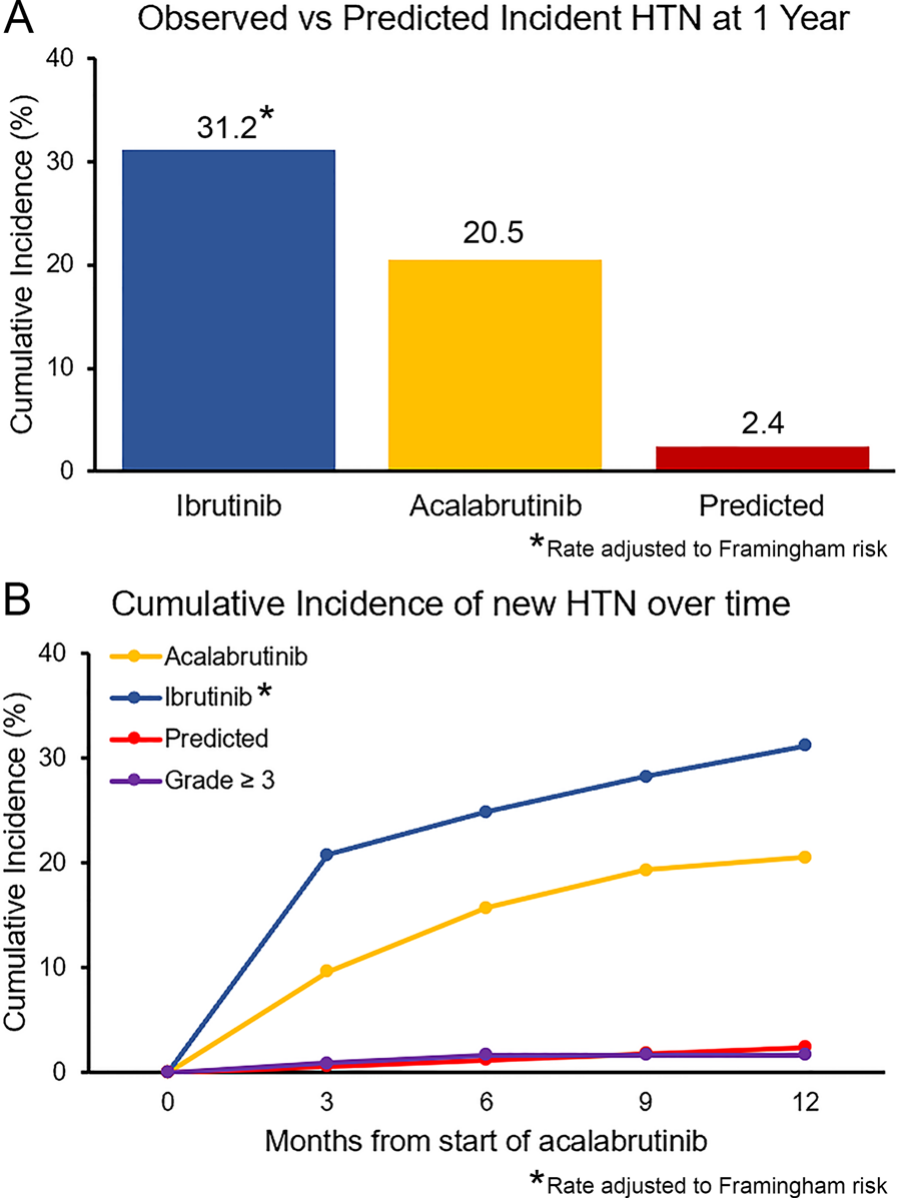
**A**. Observed versus predicted cumulative incidence of new hypertension (HTN) rates at 1 year, including population and Framingham risk-adjusted rate for ibrutinib [11, 24]. Reflects the JNC-8 HTN cutoff of 140/90 mmHg, in comparison with established HTN prediction models.^24^ Subjects without known discussion of parenteal history of HTN were assigned a value of 1 (i.e., one of two parents with HTN) in the Framingham model **B**. Observed versus predicted cumulative incidence of new HTN, including incidence of grade 3 or higher HTN, at 0, 3, 6, 9, and 12 months

Moreover, in those without baseline hypertension, 24.4% developed new hypertension using older JNC-8 cutoffs for hypertension classification. Among those patients aged 20 to 69 years without a diagnosis of diabetes (*n* = 83), the cumulative incidence of at hypertension at 1 year was 20.5%. This translated into an observed new hypertension cumulative incidence of 205 per 1,000 person-years. Compared to the Framingham risk predicted rate of 24 per 1,000 person-years, this translated into an observed-to-expected ratio of 8.5 (*P* < 0.001; Fig. 3). Yet, when compared to the previously reported Framingham-adjusted new hypertension rate at 1 year of 312 per 1,000 person-years [11] (12.9 observed-to-expected ratio), this translated into a relative risk reduction of 34.1% for acalabrutinib vs. ibrutinib for incident hypertension (RR 0.66, *P* < 0.001; Additional file 1: Table S9)*.*

## Discussion

In this evaluation of the incidence, risk factors, and ramifications of hypertension after acalabrutinib initiation, nearly 50% of patients developed new or worsened hypertension within 1 year of treatment initiation. Outside of Black ancestry and BMI, there are no other factors associated with the development of incident acalabrutinib-related hypertension. Although the rate of hypertension was markedly lower than observed with ibrutinib, the adjusted incidence was still over eightfold higher than predicted at 1 year. This relationship remained even after accounting for age and the burden of traditional cardiovascular risks. In those with early SBP elevation, the rate of other MACE was elevated. Further, blood pressure elevation control frequently required combinational therapy. Nevertheless, no antihypertensive class clearly prevented worsening hypertension. Given the growing use of second-generation BTK inhibitors, and the lack of real-world data to inform their use, these data may have important ramifications on the interpretation and management of cardiovascular risk in patients treated with these emerging therapies.

The observation of increased hypertension with acalabrutinib adds to a growing body of evidence linking BTK inhibition with blood pressure modulation. In an evaluation of ibrutinib-treated patients, initiation of ibrutinib is associated with a 71% incidence of new hypertension after treatment initiation [11]. Similarly, in a separate multicenter evaluation, ibrutinib associated with a median increase of > 13 mmHg in SBP measures within months of treatment initiation [26]. In acalabrutinib-focused studies, secondary analyses suggest that up to 40% of patients may experience increase in hypertension grade or see new hypertension [4]. Although landmark trials, including the ACE-CL-001 and ELEVATE-RR trials, have attempted to evaluate the incidence of hypertension as a secondary outcome, due to the only more recent emergence of the relevance of hypertension with BTK inhibitor therapy, without untreated controls, the measures, timing, and implementation of contemporary blood pressure definitions were not well established [1, 11, 18, 27, 28]. Furthermore, due to potential confounding nature of comorbid risk factors, understanding the true effects of therapy has remained challenging [27]. Within this study, we observed that acalabrutinib treatment results in increased rate of incident hypertension, even after accounting for confounding traditional risk factors. The establishment of these effects provides a key basis for the assessment of the vascular effects of next-generation BTK inhibition among B cell patients treated with these emerging therapies.

Blood pressure elevation is often underappreciated clinically in patients presenting for ongoing anticancer therapies [28, 29]. Due to the confounding nature of blood pressure elevation in cancer populations, particularly at times of increased stress, potential fear of negative news, and necessary focus on potentially fatal disease control, blood pressure elevations may be less well recognized in clinical care settings. However, increasingly accelerated hypertension has been noted to contribute to the unanticipated higher burden of cardiovascular events among patients treated with BTK inhibitors and other anticancer therapies [11, 28]. Blood pressure increases appeared broader and more pronounced with acalabrutinib treatment in the ASCEND trial than the standard therapy arm [30]. This was also clearly observed in the ACE-CL-001 and ACE-CL-003 phase 1b/2 studies [1, 4]. Outside of the current evaluation, post-marketing evaluations of the effects of acalabrutinib or other next-generation BTK inhibitor therapies are largely unavailable. Given the potential for differential observations after clinical dissemination in more focused investigation, and the unintended but serious consequences of cardiotoxicity, enhanced surveillance may prove beneficial.

Although reasons for these observations are not clear, acalabrutinib has reduced inhibition of alternative kinases inclusive of epidermal growth factor receptor (EGFR), extracellular signal-regulated kinase (ERK), and interleukin 2-inducible *T* cell kinase (ITK) in preclinical models compared to ibrutinib [1, 6–8]. Furthermore, in a recent preclinical AF model, acalabrutinib did not share activity with the C-terminal Src kinase (CSK), a pathway linked with AF in mice receiving ibrutinib therapy [15]. Conversely, as with ibrutinib, acalabrutinib may share indirect nitric oxide inhibition [31]. Despite differences in the degree of activation, downstream remodeling inclusive of vasoconstriction and replacement fibrosis have been postulated [11, 27]. These alterations have been recognized to drive disproportionate manifestations of cardiovascular disease in other populations [32–34]. However, with BTK inhibitors, elucidation of these pathways may require additional mechanistic and prospective studies.

### Limitations

Several limitations should be acknowledged. Due to the retrospective nature of this study, and prevalent BTKi use, no cancer-specific control was available. Follow-up was non-uniform. Similarly, the approach and timing of blood pressure acquisition were non-uniform. While antihypertensives were frequently employed in those with higher elevation, the timing and decision to initiate antihypertensive therapy was at the discretion of treating clinicians. We could not fully determine the effect of antihypertensive therapies on MACE risk or blood pressure control due to variability in treatment regimens. Similarly, the selected class and dose of therapy was not predetermined. However, the low incidence of acalabrutinib discontinuation suggests at least some efficacy of combinational standard antihypertensive care. Although we adjusted for multiple factors, it is possible that the presence of cancer increases hypertension risk [27]. Moreover, some out-of-hospital cardiac events may have gone uncaptured, despite extensive search.

## Conclusions

Patients treated with BTK inhibitors face an increased risk of cardiovascular sequelae. Treatment with acalabrutinib associates with significantly elevated risk of early onset hypertension, even after accounting for traditional risk factors. However, the degree of this hypertension is reduced compared to ibrutinib. Given the anticipated increase in acalabrutinib use, further studies evaluating the mechanisms and optimal management strategies for hypertension are needed.

## Supplementary Information

**Additional file1**: **Table S1** Long-term rates, time to development, and management of new or worsened hypertension (HTN) during acalabrutinib therapy. **Table S2** Distribution of maximum SBP increase from baseline, %. **Table S3** Development of new or worsened hypertension among patients based on concomitant obinutuzumab treatment. **Table S4** Univariable predictors for the development of new or worsened hypertension (*n*=280). **Table S5A** Multivariable predictors for the development of new or worsened hypertension, in patients not previously treated with ibrutinib (n=208). **Table S5B** Multivariable predictors for new hypertension alone (n=115).* **Table S5C** Multivariable predictors for worsened hypertension alone (n=165).*Table S6A Univariate analysis of association of single-agent baseline antihypertensive therapy (n=115) to development of worsening hypertension (HTN), excluding those on other anti-HTN medications (n=1^‡^). **Table S6B** Multivariate analysis of development of worsening hypertension (HTN), considering acalabrutinib users on single-agent baseline beta blocker therapy. **Table S6C** Multivariate analysis of development of worsening hypertension (HTN), considering acalabrutinib users on any single-agent baseline antihypertensive therapy. **Table S6D** Change in blood pressure among subjects requiring the addition of new or additional antihypertensive class within the 1^st^ year of acalabrutinib therapy. [From the 43 patients started on a new or additional antihypertensive, 17 patients saw the addition within 12 months of acalabrutinib initiation, of which 10 were treated with the addition of a single antihypertensive drug, 9 had pre- and post-antihypertensive blood pressures; another 7 (out of 17) required initiation of ≥ 2 antihypertensives and had available blood pressure measures pre- and 12 months post-initiation of the first antihypertensive added during acalabrutinib use.] **Table S7** Occurrence of major adverse cardiovascular events (MACE), by acalabrutinib-related hypertension (HTN) status. **Table S8A** Multivariable analysis for the development of MACE during acalabrutinib use, considering development of new or worsened HTN as a time-dependent covariate.*Table S8B Multivariable analysis for the development of AF during acalabrutinib use, considering development of new or worsened HTN as a non-time-dependent covariate. **Table S9** Cumulative incidence of new, predicted, and grade 3 or more HTN over time. **Figure S1** Study Cohort Diagram. From a registry of all patients with hematologic malignancies treated with acalabrutinib over a 6-year period, those with available blood pressures were included. HTN, hypertension. **Figure S2**. Risk of AF development, in relationship of observed peak SBP increase within 12 months of acalabrutinib initiation. AF, atrial fibrillation; SBP, systolic blood pressure. **Figure S3**. Cumulative incidence of disease progression or death among those remaining on acalabrutinib beyond 90 days (landmark) alone, without initial progression or death, by new or worsened hypertension status.

## Data Availability

For original data, please contact daniel.addison@osumc.edu.

## Abbreviations

BTK: Bruton’s tyrosine kinase
SBP: Systolic blood pressure
SPRINT: Systolic blood pressure interventional trial
CTCAE: Common terminology criteria for adverse events
MACE: Major cardiovascular events
AF: Atrial fibrillation
SD: Standard deviation
IQR: Interquartile range
HR: Hazard ratio
CKD: Chronic kidney disease
BMI: Body mass index
CLL: Chronic lymphocytic leukemia
ECOG: Eastern Cooperative Oncology Group
CI: Confidence interval
EGFR: Epidermal growth factor receptor
ERK: Extracellular signal-regulated kinase
ITK: Interleukin 2-inducible *T* cell kinase
CSK: C-terminal Src kinase
