# Supplementary material for: Hypertension and incident cardiovascular events after next-generation BTKi therapy initiation
Source: J Hematol Oncol. 2022 Jul 14;15:92. doi: 10.1186/s13045-022-01302-7 (PMC9281099; doi:10.1186/s13045-022-01302-7)

**Online Supplemental Material:**

**sTable 1.** Long-term rates, time to development, and management of new or worsened hypertension (HTN) during acalabrutinib therapy.

| **Final HTN status (n=280)** | **n (%)** |
| --- | --- |
| New or worsened HTN | 137 (48.9%) |
| **Months post-acalabrutinib initiation** | **Cumulative Incidence of new or worsened HTN, % (95% CI)** |
| 1 | 18.6 (14.3 – 23.3) |
| 3 | 24.3 (19.4 – 29.4) |
| 6 | 32.1 (26.8 – 37.6) |
| 12 | 39.3 (33.6 – 45.0) |
| **Group** | **Time to 50% cumulative incidence of HTN, months** |
| All patients (n=280) | 39.5 |
| No Baseline HTN (n=115) | 23.6 |
| Baseline HTN (n=165) | Not reached |
| **By baseline HTN status** | |
| **No baseline HTN (n=115)** | |
| New HTN | 62 (53.9) |
| **Baseline HTN (n=165)** | |
| Worsened HTN | 75 (45.5) |
| Worsened CTCAE grade | 38 (50.7) |
| New antihypertensive added | 33 (44.0) |
| Increased antihypertensive dose(s) | 4 (5.3) |

Abbreviations: CTCAE, Common Terminology Criteria for Adverse Events.

**sTable 2.** Distribution of maximum SBP increase from baseline, %

| **Maximum SBP Increase from Baseline, mmHg** | **Total (n=280)** | **No Baseline HTN (n=115)** | **Baseline HTN (n=165)** |
| --- | --- | --- | --- |
| ≤0 | 16.4 | 13.0 | 18.8 |
| >0-5 | 11.8 | 13.0 | 10.9 |
| >5-10 | 13.2 | 13.9 | 12.7 |
| >10-15 | 9.3 | 11.3 | 7.9 |
| >15-20 | 13.6 | 12.2 | 14.5 |
| >20-25 | 9.3 | 13.0 | 6.7 |
| >25-30 | 6.8 | 5.2 | 7.9 |
| >30-35 | 8.2 | 8.7 | 7.9 |
| >35-40 | 2.5 | 1.7 | 3.0 |
| >40-45 | 3.2 | 2.6 | 3.6 |
| >45-50 | 1.4 | 2.6 | 0.6 |
| >50 | 1.4 | 1.7 | 1.2 |

**sTable 3.** Development of new or worsened hypertension among patients based on concomitant Obinutuzumab treatment

| **Final Status** | **No or Stable HTN, (n=140), %** | **New or Worsened HTN, (n=140), %** |
| --- | --- | --- |
| Obinutuzumab | 47 (33.5) | 47 (33.5) |
| No Obinutuzumab | 93 (66.4) | 93 (66.4) |
| **Overall Statistics Based on Concomitant Obinutuzumab Therapy** | | |
|  | **Obinutuzumab** | **No Obinutuzumab** |
| Mean SBP (SD) | 13.3 (18.5) | 6.45 (20.0) |
| Median time to peak SBP increase (months) | 15 | 15 |
| Mean peak SBP increase | 14.4 | 16.8 |
| **Within 12 months of follow-up** | | |
| Mean SBP (SD) | 1.84 (12.1) | 0.76 (14.6) |
| Median time to peak SBP increase (months) | 6 | 6 |
| Mean peak SBP increase | 7.98 | 10.1 |
| **Within 3 months of follow-up** | | |
| Mean SBP (SD) | -0.96 (10.3) | 1.36 (13.7) |
| Median time to peak SBP increase (months) | 3 | 1 |
| Mean peak SBP increase | 4.63 | 6.90 |

**sTable 4.** Univariable predictors for the development of new or worsened hypertension (n=280).

| **Variable** | **Hazard Ratio** | **95% Confidence Interval** | **p-value** |
| --- | --- | --- | --- |
| Age* | 1.01 | (1.00 – 1.03) | 0.115 |
| Sex: Female vs. Male | 0.63 | (0.42 – 0.96) | 0.07 |
| Black/African-American | 1.57 | (0.43 – 5.77) | 0.494 |
| BMI* | 1.03 | (1.01 – 1.06) | **0.018** |
| BMI, n (%) | | | |
| <25 | reference | reference |  |
| 25-29.9 | 1.62 | (1.05 – 2.51) | **0.03** |
| ≥30 | 1.63 | (1.03 – 2.58) | **0.036** |
| Smoking Status: Current/Previous vs. Never | 0.87 | (0.62 – 1.22) | 0.408 |
| Prior DM | 1.56 | (0.91 – 2.67) | 0.103 |
| Prior CKD | 0.89 | (0.26 – 3.03) | 0.859 |
| Prior AF/Aflutter | 1.69 | (1.15 – 2.48) | **0.007** |
| Prior MI | 0.73 | (0.31 – 1.70) | 0.46 |
| Prior Systolic HF | 2.02 | (1.12 – 3.65) | **0.019** |
| Prior CVA/TIA | 1.76 | (0.79 – 3.95) | 0.169 |
| Hematologic Diagnosis | | |  |
| CLL | reference | reference |  |
| MCL | 1.41 | (0.37 – 5.39) | 0.617 |
| Other^‡^ | 0.24 | (0.09 – 0.67) | **0.006** |
| Baseline ECOG | | | |
| 0 | reference | reference |  |
| 1 | 1.08 | (0.77 – 1.53) | 0.645 |
| 2/3 | 0.40 | (0.10 – 1.62) | 0.2 |
| Number of Prior Anticancer Therapies | 0.91 | (0.83 – 1.00) | **0.048** |
| Concomitant Chemotherapy | 1.06 | (0.74 – 1.51) | 0.753 |
| Prior Cytotoxic Chemotherapy | 0.74 | (0.53 – 1.04) | 0.083 |
| Prior Monoclonal Antibody | 0.93 | (0.66 – 1.30) | 0.674 |
| Prior Ibrutinib Therapy | 0.78 | (0.54 – 1.15) | 0.214 |
| Prior Targeted Agent Therapy (Not Ibrutinib) | 0.77 | (0.38 – 1.55) | 0.464 |
| Prior Immunomodulatory | 0.88 | (0.50 – 1.53) | 0.647 |
| Prior Other Therapy | 1.31 | (0.73 – 2.34) | 0.371 |
| Concurrent CY3PA4 Inhibitor | 1.26 | (0.78 – 2.04) | 0.343 |
| Baseline SBP* | 0.99 | (0.98 – 1.00) | **0.027** |

Abbreviations: AF, atrial fibrillation; Aflutter, atrial flutter; BMI, body-mass-index; CKD, chronic kidney disease; CLL, chronic lymphocytic lymphoma; CVA, cerebrovascular accident; CV, cardiovascular; CYP3A4, cytochrome P450, family 3, subfamily A; DM, diabetes mellitus; ECOG, Eastern Cooperative Oncology Group; HF, heart failure; HTN, hypertension; MI, myocardial infarction; MCL, mantle cell lymphoma; TIA, transient ischemic attack. *Considered as a continuous variable. †Omnibus p-value (reflects overall variable effect). ‡Diffuse large B-Cell lymphoma, follicular lymphoma, hairy cell leukemia, graft-versus-host disease, marginal zone lymphoma, and Waldenström’s macroglobulinemia.

**sTable 5A.** Multivariable predictors for the development of new or worsened hypertension, in patients not previously treated with ibrutinib (n=208).

| **Variable** | **Hazard Ratio** | **95% Confidence Interval** | **p-value** |
| --- | --- | --- | --- |
| Age* | 1.02 | (1.00 - 1.04) | 0.096 |
| Sex: Female vs. Male | 0.67 | (0.39 - 1.14) | 0.142 |
| Black/African-American | 9.71 | (3.17 - 29.70) | **<0.001** |
| BMI* | 1.05 | (1.02 - 1.09) | **0.003** |
| Smoking Status: Current/Previous vs. Never | 0.72 | (0.48 - 1.08) | 0.116 |
| Prior DM | 1.55 | (0.65 - 3.72) | 0.32 |
| Prior CKD | 0.38 | (0.04 - 3.54) | 0.39 |
| Hematologic Diagnosis | | | |
| CLL | reference | reference |  |
| MCL | 1.20 | (0.27 – 5.38) | 0.814 |
| Other^‡^ | 0.18 | (0.06 – 0.59) | **0.005** |
| Number of Prior Anticancer Therapies | 0.95 | (0.62 - 1.47) | 0.832 |
| Baseline SBP: ≥120 vs. <120 | 0.79 | (0.50 – 1.25) | 0.309 |
| Baseline SBP, mmHg if No Baseline HTN | | |  |
| <110 | reference | reference |  |
| 110-119 | 1.25 | (0.53 - 2.96) | 0.606 |
| 120-129 | 1.73 | (0.76 - 3.90) | 0.189 |
| Baseline SBP, mmHg if Precedent Baseline HTN | | |  |
| 110-129 | reference | reference |  |
| 130-139 | 2.05 | (0.90 - 4.67) | 0.086 |
| ≥140 | 0.34 | (0.17 - 0.67) | **0.002** |

Abbreviations: BMI, body-mass-index; CKD, chronic kidney disease; DM, diabetes mellitus; SBP, systolic blood pressure. *Considered a continuous variable. †Omnibus p-value (reflects overall variable effect). ‡Diffuse large B-Cell lymphoma, follicular lymphoma, hairy cell leukemia, graft-versus-host disease, marginal zone lymphoma, and Waldenström’s macroglobulinemia.

**sTable 5B.** Multivariable predictors for new hypertension alone (n=115).*

| **Variable** | **Hazard Ratio** | **95% Confidence Interval** | **p-value** |
| --- | --- | --- | --- |
| Age** | 1.02 | (0.99 – 1.05) | 0.264 |
| Sex: Female vs. Male | 0.64 | (0.31 – 1.29) | 0.211 |
| Black/African-American | 5.44 | (1.54 – 19.16) | **0.008** |
| BMI** | 1.04 | (0.97 – 1.11) | 0.275 |
| Smoking Status: Current/Previous vs. Never | 0.72 | (0.41 – 1.28) | 0.263 |
| Prior DM | 1.22 | (0.45 – 3.27) | 0.7 |
| Prior CKD | 0.74 | (0.16 – 3.50) | 0.7 |
| Prior AF/Aflutter | 2.32 | (1.25 – 4.30) | **0.008** |
| Hematologic Diagnosis | | | |
| CLL | reference | reference |  |
| MCL | 1.92 | (0.09 – 40.29) | 0.676 |
| Other^‡^ | 0.38 | (0.07 – 2.08) | 0.265 |
| Number of Prior Anticancer Therapies | 0.85 | (0.73 – 1.00) | **0.049** |
| Baseline SBP, mmHg | | |  |
| <110 | reference | reference |  |
| 100-119 | 1.35 | (0.64 – 2.83) | 0.428 |
| 120-129 | 1.75 | (0.85 – 3.56) | 0.126 |

Abbreviations: AF, atrial fibrillation; Aflutter, atrial flutter; BMI, body-mass-index; CKD, chronic kidney disease; CLL, chronic lymphocytic lymphoma; DM, diabetes mellitus; MCL, mantle cell lymphoma; SBP, systolic blood pressure. *Reflects inclusion of variables with univariable association with new hypertension during ibrutinib use and/or established traditional hypertension risk factors. **Considered as continuous variables. †Omnibus p-value (reflects overall variable effect). ‡Diffuse large B-Cell lymphoma, follicular lymphoma, hairy cell leukemia, graft-versus-host disease, marginal zone lymphoma, and Waldenström’s macroglobulinemia.

**sTable 5C.** Multivariable predictors for worsened hypertension alone (n=165).*

| **Variable** | **Hazard Ratio** | **95% Confidence Interval** | **p-value** |
| --- | --- | --- | --- |
| Age** | 1.04 | (1.01 – 1.07) | **0.008** |
| Sex: Female vs. Male | 0.58 | (0.29 - 1.15) | 0.117 |
| Black/African-American | 5.68 | (1.26 – 25.68) | **0.024** |
| BMI** | 1.07 | (1.03 – 1.12) | **0.001** |
| Smoking Status: Current/Previous vs. Never | 0.66 | (0.40 – 1.08) | 0.095 |
| Prior DM | 1.32 | (0.44 – 4.01) | 0.621 |
| Prior CKD | 0.29 | (0.01 – 6.10) | 0.427 |
| Prior AF/Aflutter | 0.89 | (0.42 – 1.85) | 0.747 |
| Hematologic Diagnosis |  |  |  |
| CLL | reference | reference |  |
| MCL | 1.88 | (0.54 – 6.47) | 0.319 |
| Other^‡^ | 0.15 | (0.03 – 0.77) | **0.023** |
| Number of Prior Anticancer Therapies | 0.88 | (0.74 – 1.06) | 0.181 |
| Baseline SBP, mmHg | | | |
| 110-129 | reference | reference |  |
| 130-139 | 1.29 | (0.65 – 2.58) | 0.464 |
| ≥140 | 0.40 | (0.22 – 0.72) | **0.002** |

Abbreviations: AF, atrial fibrillation; Aflutter, atrial flutter; BMI, body-mass-index; CKD, chronic kidney disease; CLL, chronic lymphocytic lymphoma; DM, diabetes mellitus; MCL, mantle cell lymphoma; SBP, systolic blood pressure. *Reflects variables with univariate association with new hypertension during ibrutinib use and/or established traditional hypertension risk factors. **Considered as continuous variables. †Omnibus p-value (reflects overall variable effect). ‡Diffuse large B-Cell lymphoma, follicular lymphoma, hairy cell leukemia, graft-versus-host disease, marginal zone lymphoma, and Waldenström’s macroglobulinemia.

**sTable 6A.** Univariate analysis of association of single-agent baseline antihypertensive therapy (n=115) to development of worsening hypertension (HTN), excluding those on other anti-HTN medications (n=1^‡^).

| **Medication Class** | **Hazard Ratio** | **95% CI** | **p-value** |
| --- | --- | --- | --- |
| Beta blocker | 2.31 | (1.14 – 4.66) | **0.02** |
| ACE inhibitor/ARB | 1.08 | (0.36 – 3.28) | 0.89 |
| Calcium channel blocker | 1.63 | (0.81 – 3.28) | 0.17 |
| Diuretic† | 1.35 | (0.19 – 9.86) | 0.77 |
| Any antihypertensive | 2.41 | (1.35 – 4.30) | **0.003** |

Abbreviations: ACE, angiotensin converting enzyme inhibitor; ARB, angiotensin receptor blocker; CI, confidence interval. †Includes loop, thiazide, and potassium-sparing diuretics. ‡Clonidine, hydralazine, nitrates, and alpha-1 antagonists.

**sTable 6B.** Multivariate analysis of development of worsening hypertension (HTN), considering acalabrutinib users on single-agent baseline beta blocker therapy.

| **Medication Class** | **Hazard Ratio** | **95% CI** | **p-value** |
| --- | --- | --- | --- |
| Beta blocker | 1.44 | (0.17 – 12.3) | 0.74 |
| Black/African-American | 1.42 | (0.27 – 7.39) | 0.68 |
| Number of Prior Anticancer Therapies | 0.93 | (0.75 – 1.16) | 0.53 |
| Prior AF/Aflutter | 1.11 | (0.40 – 3.09) | 0.85 |

Abbreviations: AF, atrial fibrillation; Aflutter, atrial flutter; CI, confidence interval.

**sTable 6C.** Multivariate analysis of development of worsening hypertension (HTN), considering acalabrutinib users on any single-agent baseline antihypertensive therapy.

| **Medication Class** | **Hazard Ratio** | **95% CI** | **p-value** |
| --- | --- | --- | --- |
| Any Single-Agent Antihypertensive Therapy | 1.74 | (0.80 – 3.75) | 0.16 |
| Black/African-American | 1.74 | (0.33 – 9.25) | 0.52 |
| Number of Prior Anticancer Therapies | 0.91 | (0.71 – 1.17) | 0.47 |
| Prior AF/Aflutter | 1.00 | (0.33 – 2.98) | 1.00 |

Abbreviations: AF, atrial fibrillation; Aflutter, atrial flutter; CI, confidence interval.

**sTable 6D.** Change in blood pressure among subjects requiring the addition of new or additional antihypertensive class within the 1^st^ year of acalabrutinib therapy. [From the 43 patients started on a new or additional antihypertensive, 17 patients saw the addition within 12 months of acalabrutinib initiation, of which 10 were treated with the addition of a single antihypertensive drug, 9 had pre and post antihypertensive blood pressures; another 7 (out of 17) required initiation of ≥ 2 antihypertensives and had available blood pressure measures pre and 12 months post initiation of the first antihypertensive added during acalabrutinib use].

| **Medication Class** | **No. with single-class therapy added (n=9), %** | **Change in SBP, mmHg (Post – Pre), mean (SD)** |
| --- | --- | --- |
| Beta-blocker | 2 (22.2) | -1.32 (1.44) |
| ACE inhibitor/ARB | 2 (22.2) | -11.2 (13.9) |
| Calcium channel blocker | 4 (44.4) | +4.12 (18.4) |
| Diuretic* | 1 (11.1) | +4.25 (N/A) |
| Other anti-HTN medication† | 0 (0.0) | N/A |
| Overall | 9 (100) | -0.48 (13.9) |
| Combination^‡^ | 7 (N/A) | -3.32 (22.1) |

Abbreviations: ACE, angiotensin converting enzyme inhibitor; ARB, angiotensin receptor blocker; CI, confidence interval; N/A, not applicable. *Includes loop, thiazide, and potassium-sparing diuretics. †Clonidine, hydralazine, nitrates, and alpha-1 antagonists. ‡Subjects requiring ≥ 2 antihypertensives to be initiated during ibrutinib use.

**sTable 7.** Occurrence of major adverse cardiovascular events (MACE), by acalabrutinib-related hypertension (HTN) status.

| **Final Status** | **No or Stable HTN (n=143), %** | **New or Worsened HTN (n=137), %** |
| --- | --- | --- |
| MACE | 16 (11.2) | 25 (18.2) |
| **Cumulative Incidence of MACE (95% CI)** | | |
| **Months post-acalabrutinib initiation** | **No or Stable HTN** | **New or Worsened HTN** |
| 3 | 3.5 (1.3 – 7.5) | 1.5 (0.3 – 4.7) |
| 6 | 3.5 (1.3 – 7.5) | 1.5 (0.3 – 4.7) |
| 12 | 4.2 (1.7 – 8.4) | 5.1 (2.3 – 9.7) |
| 24 | 7.9 (4.2 – 13.2) | 8.2 (4.3 – 13.6) |
| 36 | 8.9 (4.8 – 14.4) | 12.5 (7.5 – 18.9) |
| **Median (IQR) follow-up time, months** | | |
| **Group** | **No or Stable HTN** | **New or Worsened HTN** |
| All patients | 48.8 (19.4 – 54.3) | 52.6 (30.3 – 59.8) |
| No MACE | 48.8 (21.2 – 54.7) | 50.4 (22.9 – 59.3) |

Note: Cumulative incidence analysis assumes patients develop new or worsened hypertension within 1 month of treatment initiation.

**sTable 8A.** Multivariable analysis for the development of MACE during acalabrutinib use, considering development of new or worsened HTN as a time-dependent covariate.*

| **Variable** | **Hazard Ratio** | **95% CI** | **p-value** |
| --- | --- | --- | --- |
| New/Worsened HTN versus No/Stable HTN*** | 1.12 | (0.55 - 2.30) | 0.751 |
| Age** | 1.04 | (1.00 - 1.07) | **0.037** |
| Number of Prior Anticancer Therapies | 1.08 | (0.94 - 1.24) | 0.284 |
| Concurrent CYP3A4 | 2.26 | (0.93 - 5.45) | 0.07 |
| Prior DM | 1.27 | (0.49 - 3.31) | 0.621 |
| Prior CKD | 0.96 | (0.21 - 4.30) | 0.955 |
| Prior AF/Aflutter | 3.01 | (1.44 - 6.29) | **0.003** |
| Prior CVA/TIA | 2.42 | (0.53 - 10.97) | 0.253 |
| Prior HTN | 1.23 | (0.60 - 2.52) | 0.574 |

Abbreviations: AF, atrial fibrillation; Aflutter, atrial flutter; BMI, body-mass-index; CI, confidence interval; CKD, chronic kidney disease; CVA, cerebrovascular accident; DM, diabetes mellitus; HTN, hypertension; TIA, transient ischemic attack. *MACE includes the combined outcome of AF, CHF, CVA, MI (myocardial infarction), VF/VT (ventricular fibrillation/ventricular tachycardia), and cardiovascular death during acalabrutinib use. **Considered as a continuous variable. ***HTN as time-varying.

**sTable 8B.** Multivariable analysis for the development of AF during acalabrutinib use, considering development of new or worsened HTN as a non-time-dependent covariate.

| **Variable** | **Hazard Ratio** | **95% CI** | **p-value** |
| --- | --- | --- | --- |
| New/Worsened HTN versus No/Stable HTN* | 1.29 | (0.47 – 3.54) | 0.615 |
| Age ≥ 65 | 4.07 | (1.45 – 11.40) | 0.008 |
| Prior AF/Aflutter | 4.37 | (1.85 – 10.32) | **0.001** |

Abbreviations: AF, atrial fibrillation; Aflutter, atrial flutter; HTN, hypertension. * HTN as non time-varying.

**sTable 9.** Cumulative incidence of new, predicted, and grade 3 or more HTN over time.

| **Month** | **Acalabrutinib** | **Ibrutinib*** | **Predicted** | **Grade ≥ 3** |
| --- | --- | --- | --- | --- |
| 0 | 0% | 0% | 0% | 0% |
| 3 | 9.6% | 20.8% | 0.6% | 0.9% |
| 6 | 15.7% | 24.9% | 1.2% | 1.7% |
| 9 | 19.3% | 28.2% | 1.8% | 1.7% |
| 12 | 20.5% | 31.2% | 2.4% | 1.7% |

Abbreviations: HTN, hypertension. *Rates adjusted to Framingham-predicted risk.

**sFigure 1.** Study Cohort Diagram. From a registry of all patients with hematologic malignancies treated with acalabrutinib over a 6-year period, those with available blood pressures were included. HTN, hypertension.

**sFigure 2.** Risk of AF development, in relationship of observed peak SBP increase within 12 months of acalabrutinib initiation. AF, atrial fibrillation; SBP, systolic blood pressure.


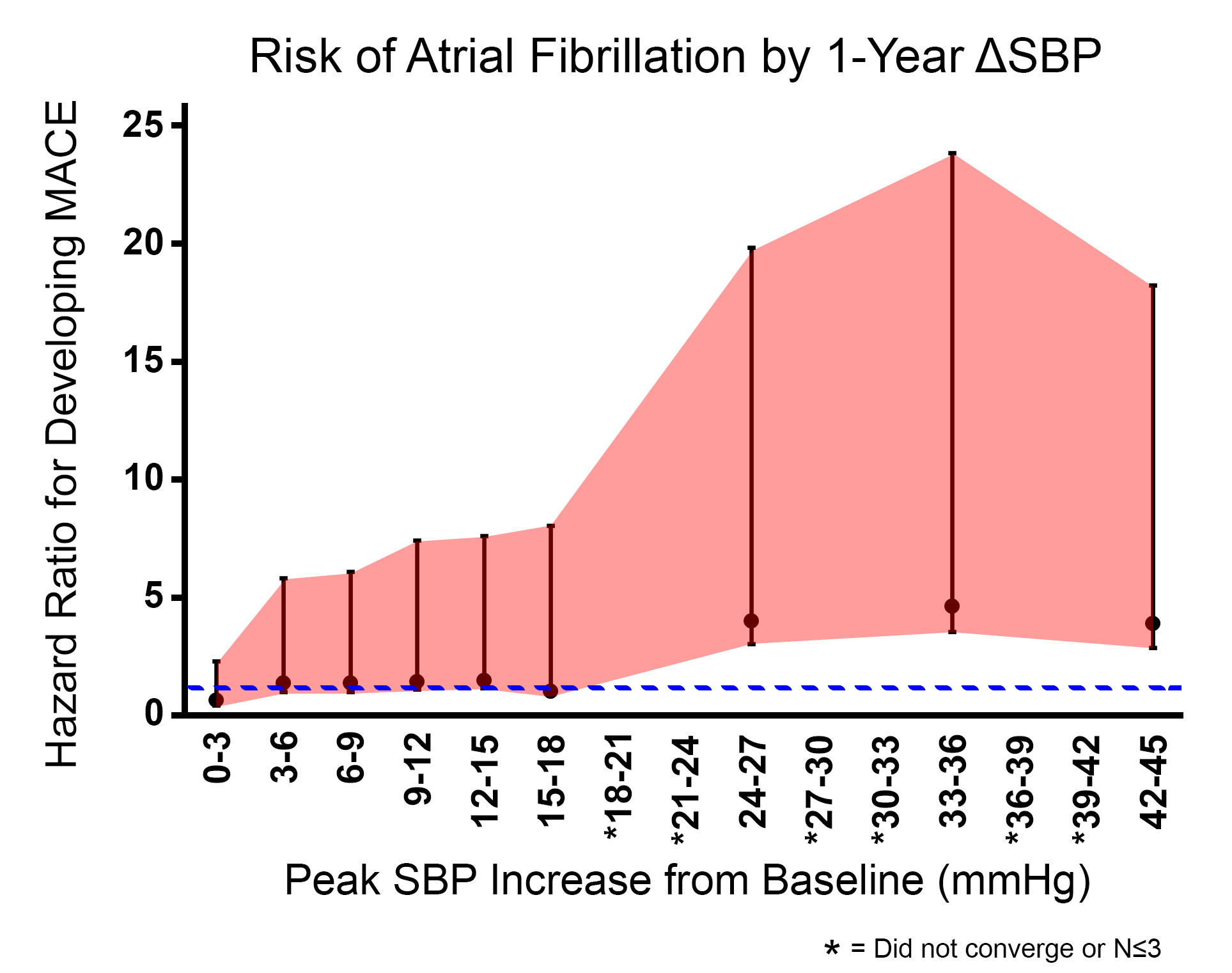


**sFigure 3.** Cumulative incidence of disease progression or death among those remaining on acalabrutinib beyond 90 days (landmark) alone, without initial progression or death, by new or worsened hypertension status.


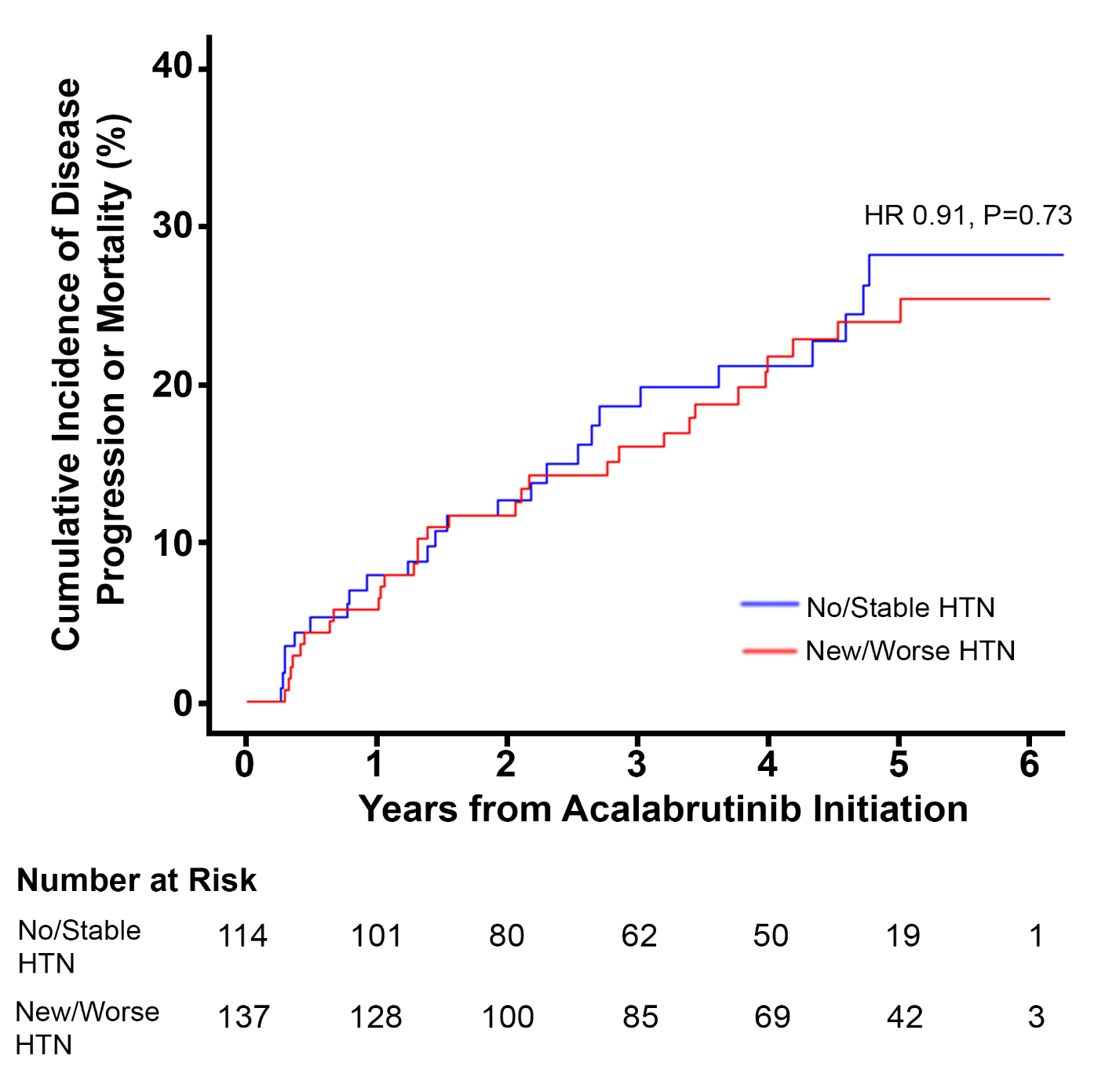

Supplement: Supplementary file 1 — Additional file1: Table S1 Long-term rates, time to development, and management of new or worsened hypertension (HTN) during acalabrutinib therapy. Table S2 Distribution of maximum SBP increase from baseline, %. Table S3 Development of new or worsened hypertension among patients based on concomitant obinutuzumab treatment. Table S4 Univariable predictors for the development of new or worsened hypertension (n=280). Table S5A Multivariable predictors for the development of new or worsened hypertension, in patients not previously treated with ibrutinib (n=208). Table S5B Multivariable predictors for new hypertension alone (n=115).* Table S5C Multivariable predictors for worsened hypertension alone (n=165).*Table S6A Univariate analysis of association of single-agent baseline antihypertensive therapy (n=115) to development of worsening hypertension (HTN), excluding those on other anti-HTN medications (n=1‡). Table S6B Multivariate analysis of development of worsening hypertension (HTN), considering acalabrutinib users on single-agent baseline beta blocker therapy. Table S6C Multivariate analysis of development of worsening hypertension (HTN), considering acalabrutinib users on any single-agent baseline antihypertensive therapy. Table S6D Change in blood pressure among subjects requiring the addition of new or additional antihypertensive class within the 1st year of acalabrutinib therapy. [From the 43 patients started on a new or additional antihypertensive, 17 patients saw the addition within 12 months of acalabrutinib initiation, of which 10 were treated with the addition of a single antihypertensive drug, 9 had pre- and post-antihypertensive blood pressures; another 7 (out of 17) required initiation of ≥ 2 antihypertensives and had available blood pressure measures pre- and 12 months post-initiation of the first antihypertensive added during acalabrutinib use.] Table S7 Occurrence of major adverse cardiovascular events (MACE), by acalabrutinib-related hypertension (HT [file 13045_2022_1302_MOESM1_ESM.docx]
